# *In vitro* human stem cell derived cultures to monitor calcium signaling in neuronal development and function

**DOI:** 10.12688/wellcomeopenres.15626.1

**Published:** 2020-02-03

**Authors:** Yojet Sharma, Sankhanil Saha, Annu Joseph, Harini Krishnan, Padinjat Raghu

**Affiliations:** 1 Cellular Organization and Signalling, National Centre for Biological Sciences - TIFR, Bangalore, Karnataka, 560065, India; 2 Brain Development and Disease Mechanisms, Institute for Stem Cell Science and Regenerative Medicine, Bangalore, Karnataka, 560065, India

**Keywords:** Stem cells, neuronal differentiation, calcium imaging, human brain disease, transcriptomics, disease in a dish

## Abstract

The development of the human brain involves multiple cellular processes including cell division, migration, and dendritic growth. These processes are triggered by developmental cues and lead to interactions of neurons and glial cells to derive the final complex organization of the brain. Developmental cues are transduced into cellular processes through the action of multiple intracellular second messengers including calcium. Calcium signals in cells are shaped by large number of proteins and mutations in several of these have been reported in human patients with brain disorders.  However, the manner in which such mutations impact human brain development *in vivo* remains poorly understood. A key limitation in this regard is the need for a model system in which calcium signaling can be studied in neurons of patients with specific brain disorders. Here we describe a protocol to differentiate human neural stem cells into cortical neuronal networks that can be maintained as live cultures up to 120 days in a dish. Our protocol generates a 2D *in vitro* culture that exhibits molecular features of several layers of the human cerebral cortex. Using fluorescence imaging of intracellular calcium levels, we describe the development of neuronal activity as measured by intracellular calcium transients during development *in vitro*. These transients were dependent on the activity of voltage gated calcium channels and were abolished by blocking sodium channel activity. Using transcriptome analysis, we describe the full molecular composition of such cultures following differentiation *in vitro* thus offering an insight into the molecular basis of activity. Our approach will facilitate the understanding of calcium signaling defects during cortical neuron development in patients with specific brain disorders and a mechanistic analysis of these defects using genetic manipulations coupled with cell biological and physiological analysis.

## Introduction

In metazoan development, extracellular cues or signals are sensed and transduced into gene expression programs that lead to cell specification and tissue/organ development. During development, these signals are sensed and transduced into cellular responses through the generation of intracellular second messengers. Changes in intracellular calcium ions [Ca ^2+^] _i_ are an important mechanism that transduces a large number of signals into cellular behaviors ^1^ including cell division, migration, shape changes and these are mediated by multiple Ca ^2+^ dependent molecular events within cells. Disruption in several elements of these Ca ^2+^ dependent processes has been linked to a number of human diseases ^2^.

The development of the brain is a complex process wherein epithelial cells that delaminate from the ectoderm form the neural tube, undergo a series of cell divisions, specifications, and movements to give rise to the cell types and organization of the adult brain. Many of these cellular processes are controlled by Ca ^2+^ signals which in addition are a key mechanism of neuronal excitability ^3^. As a result, several human brain disorders have been linked to changes in Ca ^2+^ signaling in either neurons or glial cells. For example, changes in Ca ^2+^ signaling have been linked to a number of human neurodegenerative disorders such as Alzheimer’s disease, Huntington disease, and related ataxias and Parkinson’s disease ^4^. Aberrant Ca ^2+^ signaling has also been implicated in the cellular mechanism of severe mental illness such as schizophrenia and bipolar disorder ^5^ and disorders of neuronal excitability such as epilepsy ^6^. Additionally, spurred by the development of high-throughput next-generation sequencing (NGS) technology, human genetic analyses have described mutations or variants in genes encoding components of the Ca ^2+^ signaling toolkit in human patients with brain disorders ^7^.

To date, many findings linking human brain diseases to Ca ^2+^ signaling have been inferred through the use of immortalized, stable cell lines or cellular models of rodent origin. While these have been hugely influential in driving forward the understanding of human brain disorders, there have been limited studies that have allowed such findings to be corroborated in human brain cells from patients with brain disorders. Conversely, it has been a challenge to test if variations in the DNA sequence of genes encoding components of the Ca ^2+^ signaling tool kit in human patients with brain disorders are reflected in Ca ^2+^ signaling defects in their brain cells. A principal limitation in obtaining such data is the inability to obtain brain biopsy samples from human patients and subsequently study Ca ^2+^ signaling mechanisms using live cell physiology experiments. However, the recent development of methods that allow the generation of induced pluripotent stem cells (iPSC) from somatic tissues of humans and the ability to subsequently differentiate them into neural cell types offers a solution for the need to perform live-cell physiology experiments *in vitro* from neural cells of specific genotypes or specific brain disorders ^8^. In this study, we describe protocols to differentiate human neural stem cells into cortical neurons, characterize their molecular properties and perform live cell Ca ^2+^ imaging both during neuronal development as well as in mature cultures. The use of this approach will facilitate the analysis of Ca ^2+^ signaling in human cortical neurons *in vitro* and the dissections of Ca ^2+^ signaling mechanisms that may underlie the cellular pathogenesis of human brain diseases.

## Methods

### Materials

**A) Neural Stem Cell (NSC) Culture**

*Equipment*

1. Laminar hood, CO _2_ incubator to maintain a stable environment of 37°C, 95% humidity, and 5% CO _2_.
2. Bench-top cell culture centrifuge (Plasto Crafts, Rota 4R V/FA).
3. Tissue culture treated polystyrene dishes.
4. Polystyrene conical tubes.
5. Haemocytometer (Hausser Scientific).
6. Compound phase-contrast light microscope with 4x, 10x and 20x phase objectives.

*Stock solutions and reagents*

1. Dulbecco’s Modified Eagle Medium (Life Technologies, 11995065).
2. Ham’s F-12 Nutrient Mix (Life Technologies, 11765054).
3. 50x B-27 serum-free supplement (Life Technologies, 17504044). Aliquots of 1 ml were prepared in 1.5 ml micro-centrifuge tubes and stored at -20°C.
4. 50x B-27 supplement minus Vitamin A (Life Technologies, 12587010). Aliquots of 1 ml were prepared in 1.5 ml micro-centrifuge tubes and stored at -20°C.
5. FGF-Basic (AA 1-155) Recombinant Human Protein (Thermo Fisher Scientific, PHG0261). It was reconstituted in 5 mM Tris-HCl (pH 7.54) containing 0.1% BSA at a concentration of 100 μg/ml. Aliquots of 20 μl were made and stored at -20°C. A final concentration of 20 ng/ml was used to culture and expand NSCs.
6. Heparin sodium salt (HIMEDIA, CAT NO.: 9041-08-1). Reconstituted according to manufacturer’s protocol and aliquots of 20 mg/ml were stored at 4°C.
7. Penicillin-Streptomycin (P/S) (10,000 U/mL, Life Technologies; 15140122). Aliquots of 10 ml were made and stored at -20°C.
8. 100x MEM non-essential amino acids solution (NEAA; Life Technologies; 11140050). Aliquots of 10 ml were made and stored at 4°C.
9. 100x N-2 supplement (Life Technologies; 17502048). Aliquots of 1 ml were made in 1.5 ml micro-centrifuge tubes and stored at -20°C.
10. GlutaMAX™ supplement (Life Technologies; 35050-061). Aliquots of 10 ml were made and stored at 4°C.
11. StemPro™ Accutase™ Cell Dissociation Reagent (Life Technologies, A1110501).
12. Matrigel, Growth Factor Reduced (Corning, C354277). Aliquots were prepared according to manufacturer’s instructions and stored at -80°C.
13. Human recombinant BDNF (Gibco, PHC7074). Reconstituted according to manufacturer’s protocol. Aliquots of 10 μl were stored at -20°C. A final concentration of 10 ng/ml was used during neuronal differentiation.
14. Human recombinant GDNF (Gibco, PHC7045). Reconstituted according to manufacturer’s protocol. Aliquots of 10 μl were stored at -20°C. A final concentration of 10ng/ml was used during neuronal differentiation.
15. Human recombinant IGF-1 (Gibco, PHG0078). Reconstituted according to manufacturer’s protocol. Aliquots of 10 μl were stored at -20°C. A final concentration of 10 ng/ml was used during neuronal differentiation.
16. N-[(3,5-Diflurophenyl) acetyl-]-L-alanyl-2-phenyl]gylcine-1,1-dimethlethyl ester (DAPT)—γ-secretase inhibitor (Sigma-Aldrich, D5942-5MG). It was reconstituted in DMSO at a concentration of 5 mM. Aliquots of 50 μl were stored at -20°C. A final concentration of 2 μM was used during neuronal differentiation.
17. L- Ascorbic acid 2-phosphate sesquimagnesium salt hydrate (Sigma-Aldrich, A8960). Reconstituted in sterile water to make a stock solution of 5 mg/mL, filter-sterilized and stored at -20°C.
18. Poly-L-ornithine (PLO) 0.01% (Sigma-Aldrich, P4957).
19. Laminin (LN) (Gibco, 23017015, 1 mg/ml solution).
20. PSC Cryomix freezing mixture (Thermo Fisher Scientific, A2644601). Aliquots were prepared of 10 ml and stored at -20°C.

*Primary antibodies*

**Table 1.** List of primary antibodies used in this study.

Primary antibodies	Source	Company	Catalog number	Dilution	RRID
Nestin (10C2)	Mouse (monoclonal)	Thermo Fisher	MA1-110	1:50	AB_2536821
SOX1	Rabbit (polyclonal)	Abcam	ab87775	1:200	AB_2616563
SOX2 (20G5)	Rabbit (monoclonal)	Abcam	ab92494	1:100	AB_10585428
Ki67 [B126.1]	Mouse (monoclonal)	Abcam	ab8191	1:100	AB_306346
Doublecortin	Rabbit (polyclonal)	Abcam	ab18723	1:500	AB_732011
Beta 3 Tubulin	Mouse (monoclonal)	Abcam	ab78078	1:400	AB_2256751
CTIP2 [25B6]	Rat (monoclonal)	Abcam	ab18465	1:800	AB_2064130
MAP2	Chicken (polyclonal)	Abcam	ab5392	1:1000	AB_2138153
Neurofilament	Chicken (polyclonal)	Abcam	ab4680	1:500	AB_304560
GFAP	Chicken (polyclonal)	Abcam	ab4674	1:1000	AB_304558
OLIG2 (1G11)	Mouse (monoclonal)	Thermo Fisher	MA5-15810	1:200	AB_11152534
Synapsin I	Rabbit (polyclonal)	Abcam	ab64581	1:200	AB_1281135
Synaptophysin	Rabbit (polyclonal)	Abcam	ab32594	1:500	AB_778204

**Table 2.** List of secondary antibodies used in this study.

Secondary antibodies	Species	Company	Catalog number	Dilution
Alexa 488	Goat α-Mouse IgG (Polyclonal)	Life Technology	A11001	1:300
Alexa 568	Goat α-Chicken IgG (Polyclonal)	Life Technology	A11041	1:300
Alexa 568	Donkey α-Rabbit IgG (Polyclonal)	Life Technology	A10042	1:300
Alexa 568	Goat α-Rat IgG (Polyclonal)	Life Technology	A11077	1:300
Alexa 633	Goat anti-chicken IgG (Polyclonal)	Life Technology	A21103	1:300
Alexa 633	Goat α-Rabbit IgG (Polyclonal)	Life Technology	A21070	1:300

**B) Medium**

Neuronal Expansion Medium (NEM):

a) Dulbecco’s Modified Eagle Medium/Nutrient Mixture F-12 (DMEM/F-12, 1:1 ratio)
b) 1x B27 Supplement minus vitamin A
c) 1x N2
d) 1x NEAA
e) 1x GlutaMAX
f) 100 U/ml Penicillin-Streptomycin
g) 20 ng/ml bFGF
h) 2 μg/ml Heparin Sulfate

The medium can be stored at 4°C for up to 2 weeks.

Neuronal Differentiation Medium (NDM):

a) Dulbecco’s Modified Eagle Medium/Nutrient Mixture F-12 (DMEM/F12, 1:1 ratio)
b) 1x B27 Supplement (with vitamin)
c) 1x N2
d) 1x NEAA
e) 1x GlutaMAX
f) 100 U/ml Penicillin-Streptomycin
g) 10 ng/ml BDNF
h) 10 ng/ml GDNF
i) 10 ng/ml IGF-1
j) 2 μM DAPT

The medium can be stored at 4°C for up to 2 weeks.

**C) Calcium imaging**

*Equipment*

1. Wide-field fluorescence microscope Olympus IX-83.
2. CellSens Dimensions software (Olympus, build 16686) with the necessary modules for region of interest (ROI), background subtraction, time-lapse imaging and intensity measurement.
3. 35mm glass bottom dishes prepared using in-house institute facility.

*Solutions*

1. Poly-L-ornithine, 0.01%. This was the same concentration used for the coating glass-bottom dishes.
2. Laminin (1 mg/ml). A final concentration of 10–15 μg/ml was used for coating glass-bottom dishes. The stocks were prepared as per the manufacturer’s instructions.
3. Fluo-4 AM (1 mM, Molecular probes, F14201). A final concentration of 4 μM was used for calcium imaging experiments.
4. Fura-2 AM (Thermo Fisher Scientific, F-1221). A final concentration of 4 μM was used for the store operated calcium entry (SOCE) imaging experiments.
5. TTX (HelloBio, CAS:18660-81-6). A final concentration of 10 μM was used. Working stock was prepared according to the manufacturer’s instructions.
6. Nimodipine (Sigma, N149). A final concentration of 10 μM was used. 50 mM working stock was prepared by reconstituting in methanol.
7. Ionomycin salt (Calbiochem, 407952-10MG). A 10 mM main stock of the salt was prepared by dissolving in DMSO. A final concentration of 20 μM was used for calcium imaging experiments, during calibration.
8. Pluronic acid, PF-127 (Sigma, CAS:9003-11-6, P2443-250G). A final concentration of 0.002% was used.
9. Hanks Balanced Salt Solution (HBSS): 10 mM Glucose, 10 mM HEPES, 118 mM NaCl, 4.96 mM KCl, 1.18 mM MgSO _4_, 1.18 mM KH _2_PO _4_, 2 mM CaCl _2_; pH-7.4 stored at 4°C.
10. Ethylene glycol-bis(2-aminoethylether)- *N,N,N′,N′*-tetraacetic acid (EGTA, Sigma, E3889). A stock solution of 100 mM was made and stored at room temperature. A final concentration of 4 mM was used for the SOCE experiments.

### Methods

**A. Maintenance and expansion of XCL1 Neural Stem Cells (NSCs)**

1. XCL1 NSCs (XCell Science Inc.; Novato, CA, USA; passages 7–17) was used for our experiments.
2. They were plated and expanded in NEM on 6-well plates coated with Matrigel for 1 h at 37°C/ 5% CO _2_.
3. The NSCs were routinely split in 1:3 ratio and expanded in a manner so that the cells could be utilized for experiments described in this manuscript.
4. During expansion process, the cultures were checked for mycoplasma. Following this, they were cryopreserved.

**B. Cryopreservation of XCL1 NSCs**

Frozen stocks of XCL1 NSCs were prepared by freezing of cells (~1×10 ^6^) in 1.5 ml PSC Cryomix.

**C. Differentiation of NSCs into cortical neurons**

1. NSCs (0.1–0.2 × 10 ^6^ cells) were plated on sterile 35mm glass bottom dishes.
2. These glass bottom coverslips were coated with poly-L-ornithine for 1 h at 37°C/ 5% CO _2_.
3. Coverslips were dried for 30 min. Subsequently, 10–15 μg/ml Laminin was used for a 2 ^nd^ coating and incubated for 1 h at 37°C/ 5% CO _2_.
4. The NSCs were allowed to adhere and reach 70% confluency in NEM for 1–2 days.
5. Then, NEM was withdrawn from the 35mm dishes and gently replaced with neural differentiation medium (DMEM/F12, 1X N2, 1X B27 with vitamin A, non-essential amino acids, 10 ng/ml BDNF , 10 ng/ml GDNF, 10 ng/ml IGF-1, 200 μM ascorbic acid, penicillin/streptomycin).
6. Cultures were fully refreshed with medium every 2 days during DAPT treatment (i.e. 2 weeks).
7. After 4 weeks, cultures were fully refreshed with medium every 3–4 days. ICC and calcium imaging was performed between 7 and 60 days after plating NSCs.

**D. Karyotyping of NSCs**

NSCs were grown to approximately 70% confluency in a 35 mm dish (cultured as mentioned above) and then sent to a NABL (National Accreditation Board for Testing and Calibration Laboratories-India) certified laboratory for karyotype analysis.

**E. Calculation of cell proliferation rate**

1. NSCs were seeded on a 24 well plate, 5000 cells per well, three replicates for each day.
2. Cells from 3 wells were collected every 24 hours and number of cells from each well were counted using haemocytometer. This was repeated for 7 days.

**F. Characterization of NSCs and neurons by immunofluorescent staining**

1. Cell cultures were fixed using 4% formaldehyde in phosphate-buffered saline (PBS) for 20 min and permeabilized with 0.1% TX 100 for 5 min, and incubated at room temperature for 1hr in a blocking solution of 5% BSA in PBS.
2. Primary antibodies were incubated overnight at 4°C in blocking solution, followed by incubation with secondary antibodies in blocking solution (Invitrogen) for 1 hour.
3. Confocal images were recorded by collecting a range of z-stack using an Olympus FV 3000 confocal microscope. The image stack was merged using Z-project (maximum intensity projection) tool using ImageJ 1.52n (National Institute of Health, USA, http://imagej.nih.gov/ij).

**G. Characterization of neurons by post-hoc immunofluorescent staining**

1. Post-calcium imaging, neurons were washed thrice for 10 min with HBSS buffer to wash out calcium imaging dye.
2. Neurons were fixed with either 4% PFA dissolved in PBS for 20 min or with ice-cold methanol for 5 min on bench and washed twice with PBS for 5 min each.
3. 0.1% Triton-X dissolved in PBS was used to permeabilize cells for 5 min.
4. 5% BSA or 10% Normal Goat Serum (NGS) was used for blocking for 1 hr at room temperature. BSA and NGS were dissolved in PBS.
5. Primary antibodies were used at the dilution mentioned in Table 1 and were diluted in either 5% BSA or 10% NGS. The cells were incubated with primary antibodies overnight at 4°C.
6. Following washes with PBS, secondary antibodies ( Table 2) were used at 1:300 dilution in PBS+ 5% BSA or 10% NGS and DAPI was used as a nuclear marker at a dilution of 1:1000.

**H. Quantitative real-time PCR**

*Isolation of RNA and synthesis of cDNA:*
1. Total RNA was isolated from cells in culture at specified ages using TRIzol (Ambion, Life Technologies, catalog no. 15596018) as per manufacturer’s instructions.
2. The total RNA was quantified using a Nanodrop 1000 spectrophotometer (Thermo Fisher Scientific). Three biological replicates for isolated RNA from each sample were used for the validation of gene expression by quantitative real time PCR (qRT-PCR).
3. Approximately 1 μg of total RNA was used per sample for cDNA synthesis.
4. The RNA was treated with 1U of DNase I (amplification grade, Thermo Fisher Scientific, catalog no. 18068-015) in a reaction mixture of 45.5 μl containing 10 mM DTT and 40U of RNase inhibitor (RNaseOUT, Thermo Fisher Scientific, catalog no.10777-019). The reaction mixture was incubated at 37°C for 30 min followed by heat inactivation at 70°C for 10 min.
5. For the synthesis of cDNA, 200U of Superscript II Reverse Transcriptase (Invitrogen, catalog no. 18064-014) was added to the reaction along with 2.5 μM random hexamers, and 0.5 mM dNTPs in a final volume of 50 μl. The reaction mixture was incubated at 25°C for 10 min, followed by 42°C for 60 min and heat inactivated at 70°C for 10 min (ProFlex PCR Systems, Life Technologies).
*qRT-PCR analysis:*
6. Quantitative qRT-PCR analysis was performed in a total volume of 10 μl with Power SYBR Green Master mix (Applied Biosystems, catalog no.4367659) on an Applied Biosystems ViiA7 system.
7. qRT-PCR analysis was performed with diluted cDNA samples and primers for genes of interest and GAPDH was used as an internal control. The primers used for qRT-PCR were designed using Primer-BLAST, NCBI and their details are provided in Table 3.
8. Technical triplicates were performed for each qRT-PCR reaction. As a control, a reaction with no reverse transcriptase was also set up.
9. The reaction was run using the following conditions: 50°C for 2 minutes, 95°C for 10 minutes, followed by 40 cycles of 95°C for 30 seconds (denaturation), 60°C for 30 seconds (annealing) and 72°C for 45 seconds (extension).
10. A dissociation curve was generated after amplification to distinguish the actual PCR products from primer dimers and non-specific products under the following conditions: 95°C for 15 seconds, 60°C for 1 minute, 95°C for 30 seconds and 60°C for 15 seconds.
11. The C _t_ values obtained for different genes were normalized to those of GAPDH from the same sample. The relative expression levels were calculated using ∆C _t_ method, using the formula 2 ^-∆Ct^.
12. One-way ANOVA was used for Statistical analysis using GraphPad Prism 5.0 software, and p < 0.05 was considered significant.

**Table 3.** List of qRT-PCR primers.

Gene name	NCBI Reference (mRNA)	Forward primer (5'-3')	Reverse primer (5'-3')
*GAPDH*	NM_002046.6	TGCACCACCAACTGCTTAGC	GGCATGGACTGTGGTCATGAG
*CTIP2/BCL11B*	NM_138576.3	CAGGAGAACATTGCAGGGCCG	GGCACGCAGAGGTGAAGTGA
*CUX1*	NM_181552.3	TGCAGAAAACTGCAGAGCCG	TTTAAGGCAGGGTCGAGGGC
*DCX*	NM_000555.3	TGACTCAGCAAACGGAACCT	CGAGTCCGAGTCATCCAAGG
*GAD67/GAD1*	NM_000817.2	AAGCTACACAAGGTGGCTCC	CATCCGGAAGAAGTTGGCCT
*GFAP*	NM_002055.4	AGAACCGGATCACCATTCCC	CACGGTCTTCACCACGATGT
*MAP2*	NM_002374.3	GCGCCAATGGATTCCCATAC	CAGACACCTCCTCTGCTGTT
*NEFH*	NM_021076.3	GGCACTGAAAAGCACCAAGG	CTGCTGAATGGCTTCCTGGTA
*NEUN/RBFOX3*	NM_001082575.2	ACGATCGTAGAGGGACGGAA	TTTAGCTTCCAGCCGTTGGT
*OLIG2*	NM_005806.3	GGTGCGCAAGCTTTCCAAGA	GATCTCGCTCACCAGTCGCT
*PSD95/DLG4*	NM_001365.4	GAGAGTCAGAAATACCGCTACC	CCCGTTCACCTGCAACTCAT
*SYN*	NM_006950.3	GTGGACACGTGCTCAGAGAT	AGGAACCCACCACCTCAATG
*SYP*	NM_003179.2	TGGGGACTACTCCTCGTCAG	GTGGCCAGAAAGTCCAGCAT
*TUBB3*	NM_001197181.1	ATCTTTGGTCAGAGTGGGGC	CTGCAGGCAGTCGCAGTTTT
*VGLUT1/SLC17A7*	NM_020309.3	AGCTGGGATCCAGAGACTGT	CCGAAAACTCTGTTGGCTGC
*GLS*	NM_014905.4	GCTATGGACATGGAACAGCG	TATTCCACCTGTCCTTGGGGA
*SATB2*	NM_001172509.1	AAGGCCGTGGGAGGTTTGAT	TTTCCGCACCAGGACAAACT
*STIM1*	NM_001277961.1	CCTGTGGAAGGCATGGAAGT	GTCCCTGTCATGGTGGTGTT
*STIM2*	NM_001169118.1	GCATGTCACTGAGTCCACCA	TCTCTGTGCAGATGGCTGTG
*ORAI1*	NM_032790.3	GGTCAAGTTCTTGCCCCTCA	CGTTGAGCTCCTGGAACTGT
*ORAI2*	NM_001126340.3	ATCCACTTCTACCGCTCCCT	CATCTGACAAATCCCCGCCT
*ORAI3*	NM_152288.3	AGAGTGACCACGAGTACCCA	TGTGGATGTTGCTCACAGCT
*ITPR1*	NM_001099952.2	TGCCCAAAAGCAGTTCTGGA	TACGGTCCCCAGCAATTTCC
*ITPR2*	NM_002223.4	CTGAGAAGCGAGGGTGACAA	CCAGCTGGTGTTGCAATTGA
*ITPR3*	NM_002224.4	GTTCCTGACGTGTGACGAGT	AAGCGGTACAAGCCATTCCA
*ATP2A2/SERCA2*	NM_001681.4	GCAATTGTGGGTGTATGGCA	ATCCGCTGCACACTCTTTCT
*ATP2A3/SERCA3*	NM_005173.4	TCGAGGCCCTGAAGGAGTAT	CAGCGTGGTGGACTTGATCT

**I. Calcium imaging to measure transients**

*Cell preparation and dye loading:*
1. 35mm glass-bottom coverslip dishes were UV sterilized and coated with PLO/Laminin before plating cells. 0.01% PLO was coated for 1hr followed by Laminin coating. Following each coating step, the dishes were air-dried.
2. 0.1–0.2 million cells were seeded in 35mm glass-bottom coverslip dishes. Prior to imaging, NDM was aspirated out of the dishes and the cells were washed twice (each for 5 min) with HBSS (10 mM HEPES, 118 mM NaCl, 4.96 mM KCl, 1.18 mM MgSO _4_, 1.18 mM KH _2_PO _4_, 2 mM CaCl _2_; pH-7.4), supplemented with 10 mM Glucose.
3. Calcium imaging was carried out on 7, 14, 21, 30 and 45 days *in-vitro* (DIV) neurons. Prior to dye loading, 1mM of Fluo-4/AM was diluted to 4 μM in calcium imaging buffer; to avoid compartmentalization of the dye, PF-127, a permeabilizing agent, was diluted to 0.002% in the calcium imaging buffer.
4. Cells were incubated for 30-45 mins in dark at room temperature with 4 μM Fluo-4 AM.
5. Following dye loading, the cells were washed again with the calcium imaging buffer thrice, each wash for 5 mins. Finally, cells were incubated for an additional 20 min at room temperature to facilitate de-esterification.
*Imaging:*
6. The imaging parameters were set before initiating imaging. Ca ^2+ ^imaging was performed for 10 min with a time interval of 1second using a 20X objective of wide-field fluorescence microscope Olympus IX-83.
7. A four-minute long baseline measurement was recorded to visualize calcium transients, followed by the addition of TTX to abolish calcium transients for another 4 mins.
8. Lastly, ionomycin was added to the dish followed by 5 mM MnCl _2 _solution for calibration.
*Image analysis:*
9. Image analysis was performed using the CellSens Dimensions; a region of interest (ROI) was drawn manually around each cell and the time-lapse acquisition mode of the software was used to track fluorescence changes over time.
10. The raw fluorescence intensity values from each neurons was normalized to the first fluorescence intensity signal of the baseline recording.
11. A first-derivative filter was then used to threshold and identify calcium transients.
12. A calcium event for our experiments was defined by a positive derivative value of 2×SD + average of normalized intensity in regions where devoid of transients.
13. Number of spikes and amplitude from individual neurons were measured manually from first-derivative filter traces.
14. The analyses were plotted on Graph Pad Prism 7.

**J. Calcium imaging for SOCE experiments**

*Dye loading:*
1. The NSCs and the neurons were grown on Poly-L-Lysine (PLL)/ Laminin coated coverslip dishes (as described before).
2. Prior to imaging, the cells were washed twice with HBSS (10 mM HEPES, 118 mM NaCl, 4.96 mM KCl, 1.18 mM MgSO _4_, 1.18 mM KH _2_PO _4_, 2 mM CaCl _2_; pH-7.4), supplemented with 10 mM Glucose.
3. The cells were then loaded with 4 μM of the dual-excitation single emission ratiometric Ca ^2+^ indicator Fura-2 AM (Acetoxymethylester, Invitrogen) and incubated in dark at room temperature for 30 min. Fura-2 AM was dissolved in the HBSS buffer, containing 0.002% Pluronic F-127.
4. After the loading, excess dye was washed off by washing the cells thrice with HBSS and cells were incubated for an additional 20 min at room temperature before imaging for de-esterification.
5. Just before imaging, HBSS buffer was replaced by ‘zero Ca ^2+^ HBSS’ (10 mM HEPES, 118 mM NaCl, 4.96 mM KCl, 1.18 mM MgSO _4_, 1.18 mM KH _2_PO _4_, 10 mM Glucose; pH-7.4).
*Imaging:*
6. Ca ^2+ ^imaging was performed for 17 min using a 40X objective (N.A. 0.75) of wide-field fluorescence microscope Olympus IX-83.
7. The cells were alternately illuminated at 340 nm and 380 nm using band-pass filters and the fluorescence emission at 510 nm was acquired using an EM-CCD camera (Evolve 512 Delta, Photometrics). 340 nm/380 nm ratio images were recorded at every 5 s intervals.
8. Basal cytosolic Ca ^2+ ^was recorded in ‘zero Ca ^2+^ HBSS’ for 12 frames.
9. Following this, 10 μM Thapsigargin (TG, Invitrogen) was added to the cells for inducing Ca ^2+ ^release from the endoplasmic reticulum store; images were acquired every 5 s for 84 frames.
10. 2 mM CaCl _2_ was added to the extracellular buffer to induce SOCE after which images were recorded every 5 s for 60 frames.
11. Finally, 20 μM Ionomycin (Calbiochem) was added and 24 frames were recorded for obtaining the maximum fluorescence intensity.
12. Subsequently, 4 mM EGTA (containing 0.01% Triton-X-100) was added for chelating all the cytosolic Ca ^2+ ^and 24 frames were recorded for the minimum florescence intensity for each cell.
*Image analysis:*
13. Image analysis was performed using the CellSens Olympus software; a region of interest (ROI) was drawn manually around each cell and the time-lapse acquisition mode of the software was used to track fluorescence changes over time.
  The emission intensities corresponding to 340 nm and 380 nm excitations were used to calculate the 340/380 ratio for each cell across all time points. The [Ca ^2+^] _i_ was estimated using the Grynkiewicz equation ^9^ as follows:
  $$\left[ca^{2+}]\;nM=K_{d}\times SF\times (\frac{R-R_{min}}{R_{max}-R}\right)$$
  where R _min_ and R _max_ correspond to the minimum 340/380 ratio and maximum 340/380 ratio respectively. 225 nM was taken as K _d_ for Fura-2 in human cells and SF (scaling factor) was calculated for each cell by dividing the fluorescence emission intensity at Ca ^2+^ free form with the fluorescence emission intensity at Ca ^2+^ bound form of the dye after excitation at 380 nm.

**K. Library preparation, sequencing and RNA-Seq data analysis**

*Library preparation:*
1. Total RNA was isolated from XCL1 NSCs, 45 DIV and 60 DIV neurons using TRIzol as per manufacturer’s instructions.
2. Total RNA was quantified using Qubit4 dsDNA HS Assay Kit (Thermo Fisher Scientific, catalog no.Q32854) and run on a Bio-analyzer chip (Agilent High Sensitivity DNA Chip, catalog no.5067-4626) for ensuring the integrity.
3. Approximately 150 ng of total RNA (RIN values > 9) was used per sample to prepare libraries using the NEBNext Ultra II Directional RNA Library Prep Kit for Illumina (New England Biolabs, catalog no. E7760L) following the NEB Next Poly(A) mRNA Magnetic Isolation Module (catalog no.E74906).
4. The prepared libraries were then run on a Bio-analyzer using Agilent High Sensitivity DNA Chip to check their size.
5. The libraries were then quantified by qRT-PCR and sequenced on Illumina Hiseq 2500 sequencing platform using 2x100 bp sequencing format (Illumina).
6. Biological triplicates were performed for RNA isolated from XCL1 NSCs while duplicates were performed for RNA isolated from 45 DIV and 60 DIV neurons. All the seven samples were run in a 2X100 paired end Single Flow Cell.
*Differential expression of genes:*
1. The paired-end reads (50 million reads per sample), quality checked and processed (adapter removal and trimming) were obtained from RNA sequencing as mentioned above.
2. The quality of the reads was further verified using FastQC v0.11.8 ^10^ quality check.
3. The fasta sequence file and .gtf file for human genome were downloaded from the Ensemble database.
4. The reads were aligned to the human genome. The reads for each sample (NSC in triplicate, 45 DIV and 60 DIV neurons in duplicates) were mapped on the human genome (Genome Reference Consortium Human Build 38 Organism: *Homo sapiens* (human)).
5. RSEM v1.3.1 ^11^ was used for preparing the reference files and for mapping the reads. The reads were mapped to the reference genome and a count file containing counts of reads for each gene was obtained using rsem-calculate-expression module.
6. The DESeq 1.38.0 ^12^ method was used for calculating the log _2_ fold change (log _2_FC) from the counts for each gene.
7. The genes with a log _2_FC of +1.5 and greater and significant p-value and FDR (<0.05 and <0.05 respectively) were considered to be upregulated while the genes with a log _2_FC of -1.5 and lesser with significant p-value and FDR (<0.05 and <0.05 respectively) were considered to be downregulated.
8. A list of genes involved in calcium signalling in neurons was collated from ^13^ - A total of 109 genes were selected to understand the variation in calcium signalling between NSCs and DIV45 and DIV60 neuron samples. The differential expression of these genes based on their log _2_FC values was analysed and represented in the form of heatmap.

## Results

### Characterization of NSC

**Figure 1.**
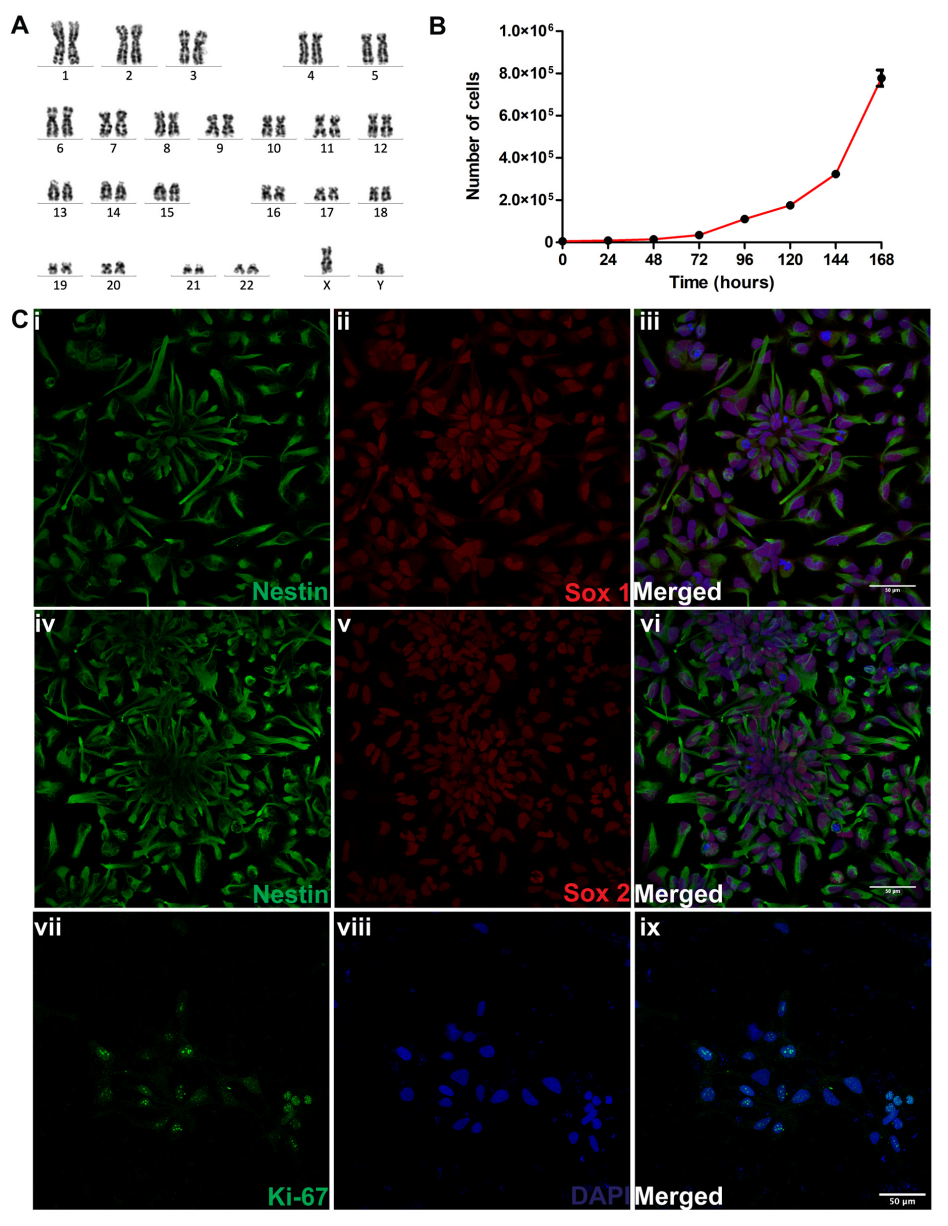
Characterization of XCL-1 neural stem cells (NSCs). ( **A**) Cytogenetic characterization of NSCs by karyotyping. Image of chromosome from a metaphase spread of XCL1 showing normal number and banding pattern of each of the 22 pairs of autosomes, X and Y chromosomes. ( **B**) Proliferation curve of XCL1, with the values represented as mean ± SEM of 3 independent measurements. Y-axis depicts the number of cells and the X-axis, age in hours ( **C**) Confocal images of XCL-1 showing the expression of (i–iii) Nestin/SOX1. Confocal images of (iv–vi) Nestin/SOX2 and (vii–ix) Ki-67/DAPI. The NSCs were regularly observed in their typical neural rosette morphology. The confocal images in this panel were brightness adjusted using ImageJ for better visualization. Scale bar 50 μm.

We characterized the starting culture of NSC using a number of approaches. The cell line that we used, XCL-1 was originally derived from CD34+ cord blood cells of a normal newborn male subject. Although purchased commercially, we karyotyped them during this study and found them to have normal karyotype ( Figure 1A); karyotyping was repeated every 10 passages. XCL-1 NSCs were cultured on a plastic surface coated with a commercially available extracellular matrix (Matrigel-Corning). They attached readily to this substratum and had a doubling time of ca. 24 hrs ( Figure 1B). Ki-67, a proliferative marker, was also used to validate the proliferation of NSCs ( Figure 1C vii, ix) and we observed 32.15% of cells positive for Ki-67 (91 out of 283 cells were positive for Ki-67). Immunocytochemistry analysis showed that these NSCs expressed the widely used NSC markers, Nestin, SOX1, and SOX2 ( Figure 1 C i-vi). For example, in the case of Nestin, 98% of the cells in the culture showed expression of this protein marker.

### Differentiation of NSC into neurons *in vitro*

**Figure 2.**
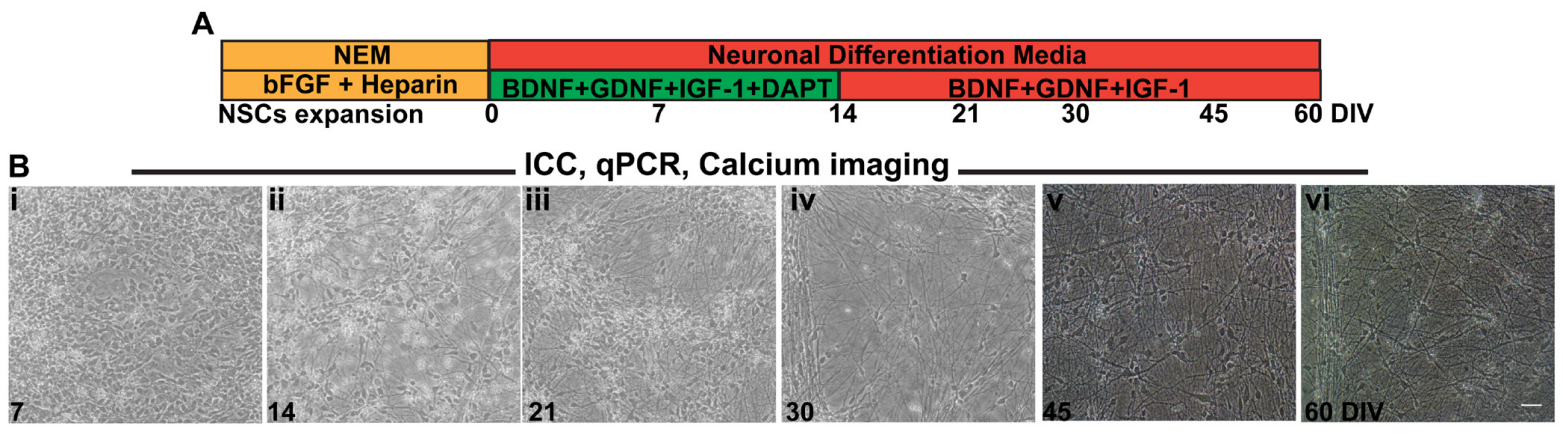
Neuronal differentiation of XCL-1 neural stem cells (NSCs). ( **A**) Schematic representation of neuronal maintenance and differentiation. Top row shows the name of the medium and the bottom row indicates the growth factors and other small molecules added in each case. The age of the cultures is shown below in days *in vitro* (DIV). ( **B**) Phase contrast images of NSC and cultures during differentiation. At each time point, cells were characterized using immunocytochemical staining (ICC), qRT-PCR, and calcium imaging. (i) 7 DIV(ii) 14 DIV (iii) 21 DIV (iv) 30 DIV (v) 45 DIV and (vi) 60 DIV. Some cell death was observed in 7 DIV which gradually decreased over days in culture; (ii) By 14 DIV, bipolar neuronal morphologies are observed (iii–vi) From 21 DIV- 60 DIV cells start to cluster together in clumps and spread out as a mat. Scale bar 50 μm.

We obtained human neuronal cultures *in vitro* by differentiating NSCs. The NSCs were expanded and re-plated on PLO-laminin coated coverslip dishes in Neural Expansion Medium. The neuronal differentiation protocol has been outlined in Figure 2A. Initiation of cortical neuronal patterning is achieved by gradually withdrawing bFGF and heparin and addition of neurotrophic factors BDNF, GDNF, IGF-1 and the γ-secretase inhibitor DAPT that inhibits Notch signaling. Over 7 days *in vitro* (DIV), the NSCs began to exhibit reduced proliferation and also show cell death ( Figure 2Bi-vi). They also form small aggregates and established elongated spindle processes. Such cultures were maintained for upto a maximum of 120 DIV and characterized for the expression of transcripts and proteins to establish the molecular process of neuronal differentiation and the identity of the cells in culture.

**Figure 3.**
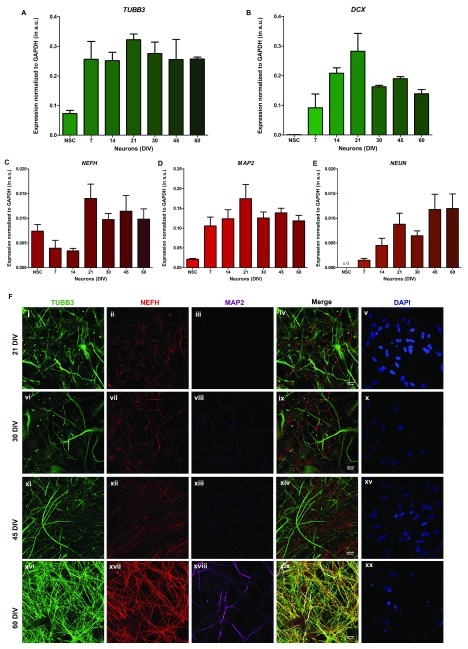
Characterization of neurons differentiating from neural stem cells. ( **A**) to ( **E**) Relative mRNA expression levels of early and late neuronal markers at stages of neuronal differentiation obtained from qRT PCR. The figures A and B illustrate the relative expression of early neuronal markers *TUBB3* and *DCX*, and figures C, D and E shows *NEFH*, *MAP2* and *NEUN* respectively. X-axis shows the age of the cultures in days *in vitro* (DIV). The expression levels of each transcript have been normalized with *GAPDH* as endogenous control and the values represented in the terms of 2 ^-∆Ct^, mean ± SEM. One-way ANOVA was used for statistical analysis, and p < 0.05 was considered significant. ( **F**) Immunofluorescence images (maximum intensity projections) showing stages of neuronal differentiation from neural stem cells. The cells were stained with the neuronal markers TUBB3/β-tubulin III (green), NFEH/neurofilament heavy polypeptide (red) and MAP2 (magenta) followed by counterstaining with DAPI (blue). The figures i to v represent 21 DIV, vi to x represent 30 DIV, xi to xv represent 45 DIV and xvi to xx represent 60 DIV. Scale bar: 20 μm.

The differentiation of NSC into cortical neurons follows a number of stages including early neuronal differentiation, the formation of neurons typical of each of the six layers of the human cerebral cortex and the development of synaptic connections. We monitored these events by measuring the expression of transcripts for genes known to be expressed at each of these stages of development. Gene expression analysis was done using qRT-PCR on neuronal cultures at 7, 14, 21, 30, 45 and 60 DIV. This analysis revealed a gene expression pattern consistent with the known steps of cortical neuronal differentiation. We found that the expression of the immature neuronal marker β3-tubulin (TUBB3) starts increasing from 7 DIV onwards ( Figure 3A). Doublecortin (DCX), a microtubule binding protein required for migration of neural progenitors and associated with X-linked lissencephaly in humans ^14, 15^ increased over the first 21 DIV after which its transcripts declined ( Figure 3B). The decline in DCX expression at DIV21 was associated with a continued increase in the expression of the mature neuronal marker NeuN ( Figure 3E) that is encoded by *RBFOX3*, a member of the Foxo family of transcription factors; this is consistent with the previously reported expression pattern of NeuN *in vivo* ^16^. In addition, we found that expression of the mature neuronal markers microtubule-associated protein 2 ( *MAP2*) and Neuronal Heavy polypeptide ( *NEFH*) shows delayed temporal expression compared to β3-tubulin transcripts ( Figure 3D, E). A parallel immunocytochemistry study revealed that TUBB3 increases progressively from 21 DIV onwards ( Figure 3F i, vi, xi, xvi). By contrast, although expression of the NEFH protein was detected from 21 DIV, enhanced expression was only seen by 60 DIV ( Figure 3F xvii); this was also true for MAP2 ( Figure 3F xviii).

### Development of human cortical layer cell types

**Figure 4.**
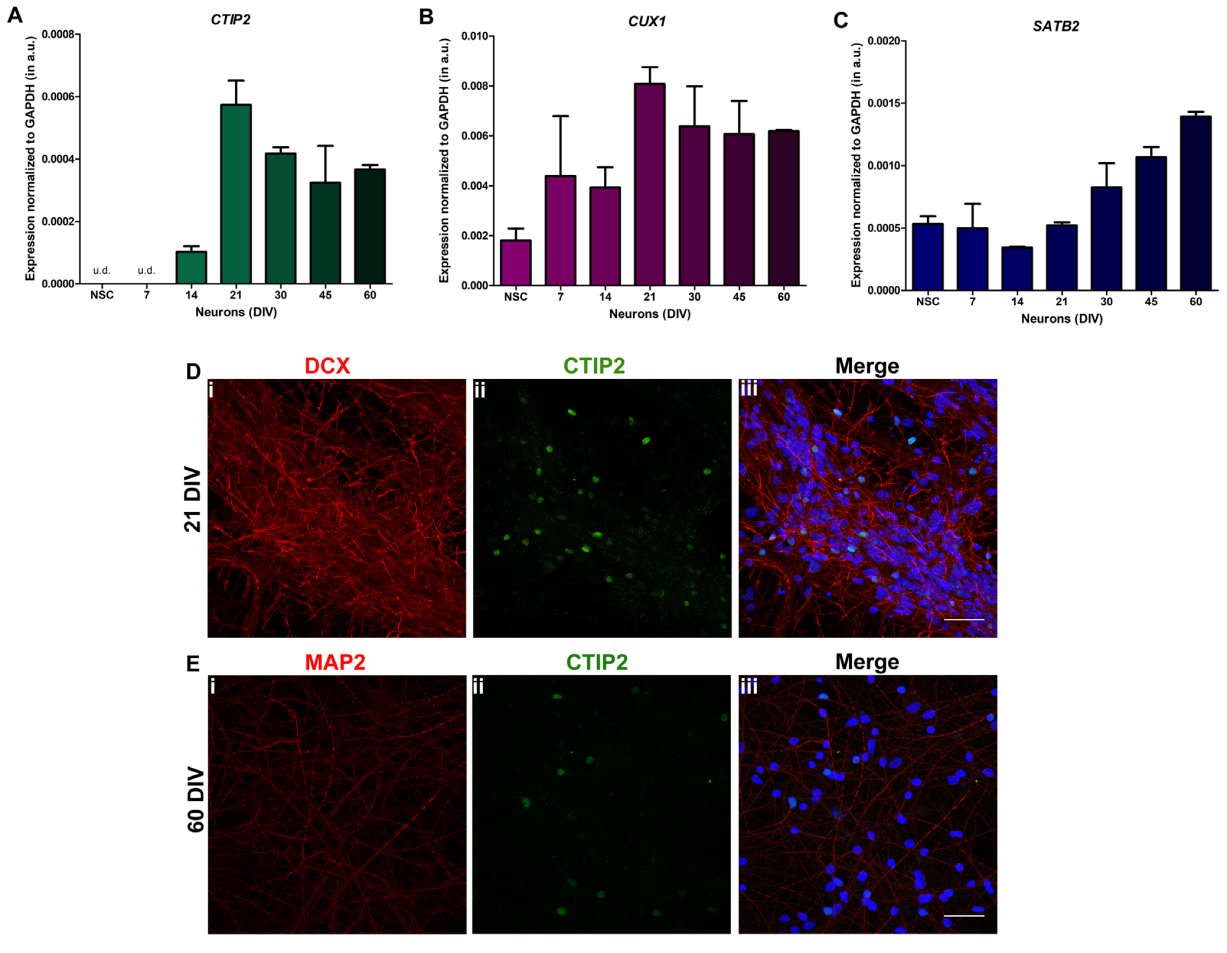
Expression of cortical layer neuronal markers ( **A**) to ( **C**) Relative mRNA expression levels of cortical neuronal layer markers *CTIP2*, *CUX1* and *SATB2* at various stages of neuronal differentiation obtained from quantitative real time PCR (qRT PCR). The expression levels have been normalized with *GAPDH* as endogenous control and the values represented in the terms of 2 ^-∆Ct-^Y-axis, mean ± SEM. One-way ANOVA was used for statistical analysis, and p < 0.05 was considered significant. ( **D**) Representative images of immature neuronal marker DCX (red) and layer-V marker CTIP2 (green) observed in culture from 21 days *in vitro* (DIV) onwards. ( **E**) 60 DIV neurons were immunostained with mature neuronal marker MAP2 (red) to validate presence of mature neurons expressing CTIP2 (green).

The development of the human cerebral cortex is choreographed through the expression of unique transcription factors typical of and necessary for specific layers of the developing cortex. To test the extent to which our *in vitro* cultures recapitulate the cell types in these cortical layers, we measured the expression of these transcription factors during *in vitro* development. Cut like homeobox 1 (CUX 1) is a transcription factor expressed in and required for dendritic morphogenesis in Layer II-III cortical neurons ^17^. We found that the expression of *CUX1* progressively rose from 7 DIV to 21 DIV after which it remained elevated ( Figure 4B). Likewise, the expression of *CTIP2* a transcription factor typical of layers V-VI was upregulated at 21 DIV and remained elevated thereafter ( Figure 4A). Immunolabelling with a CTIP2 antibody revealed the expression of CTIP2 protein in the nucleus of a subset of neurons in our 21 DIV and 60 DIV cultures ( Figure 4 D,E). We also quantified the expression of *SATB2*, a chromatin remodeling factor that regulates the specification of neurons in cortical layer II-IV ^18, 19^ and found that its expression is upregulated from 21 DIV onwards and then increased progressively ( Figure 4C). Thus, our *in vitro* differentiation protocol recapitulates key molecular markers of human cerebral cortex neurogenesis as previously reported *in vivo*.

### Expression of synaptic transmission elements *in vitro*

**Figure 5.**
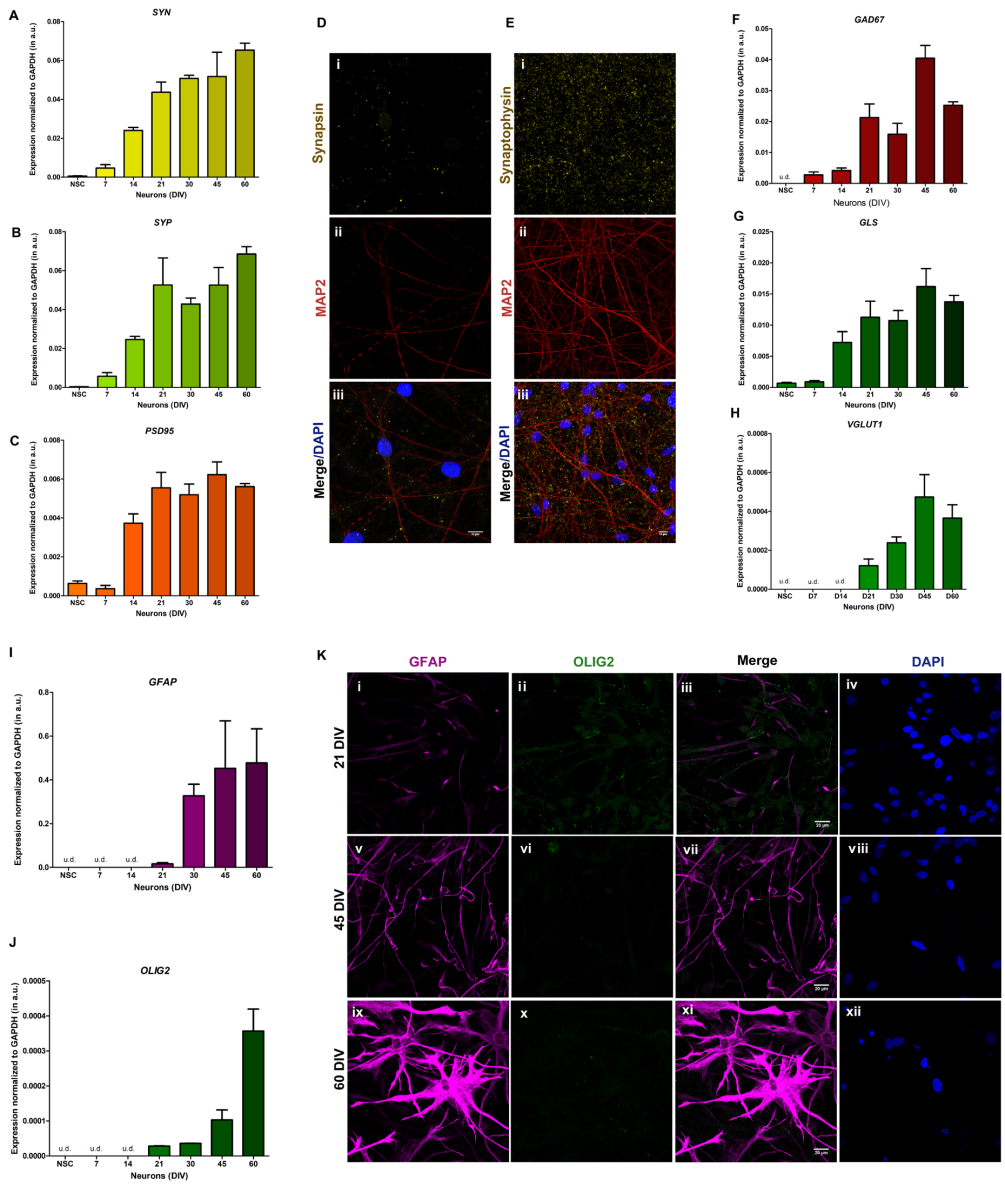
Expression of synaptic and glial markers during *in-vitro* differentiation. ( **A**) to ( **C**) Relative mRNA expression levels of synaptic markers *SYN*, *SYP* and *PSD95* at stages of neuronal differentiation obtained from quantitative real time PCR (qRT PCR). The expression levels have been normalized with *GAPDH* as endogenous control and the values represented in the terms of 2 ^-∆Ct-^Y-axis, mean ± SEM. ( **D**) Presence of presynaptic protein Synapsin 1 (yellow) and ( **E**) synaptic vesicle protein Synaptophysin (yellow) in MAP2 (red) positive neuronal cells. The nuclei of the cells were observed by staining with DAPI (blue). ( **F**) to ( **H**) qRT PCR results of relative mRNA expression in GABAergic neuronal marker *GAD67*, and glutamatergic neuronal markers *GLS* and *VGLUT1* respectively. ( **I**) and ( **J**) Relative mRNA expression levels of astrocyte and oligodendrocyte markers *GFAP* and *OLIG2* respectively at different stages of neuronal differentiation, as obtained from qRT PCR. The expression levels of all the genes have been normalized with *GAPDH* as endogenous control and the values represented in the terms of 2 ^-∆Ct^, mean ± SEM. One-way ANOVA was used for statistical analysis, and p < 0.05 was considered significant. ( **K**) Immunofluorescence images (maximum intensity projections) showing expression of GFAP (magenta) and OLIG2 (green) at stages of neuronal differentiation from neural stem cells. The cells were stained with astrocyte marker GFAP and oligodendrocyte marker OLIG2, followed by counterstaining with DAPI (blue). The figures i to iv represent to 21 DIV, v to viii represent 30 DIV, ix to xii represent 45 DIV and xiii to xvi represent 60 DIV. Scale bar: 20 μm.

We also measured the expression of transcripts for key proteins involved in the organization and function of the synapse. These include the presynaptic protein synapsin (SYN) synaptophysin (SYP), and the post-synaptic density protein PSD-95. Levels of transcripts for these molecules were found to increase from about 21 DIV and peak between 45 to 60 DIV ( Figure 5A–C). Immunofluorescence labelling also revealed the presence of punctate labelling for both SYN and SYP in 60 DIV neurons ( Figure 5 D, E). The cerebral cortex consists of neurons that are both excitatory glutamatergic and inhibitory GABAergic ( *γ*-aminobutyric-acid) with respect to neurotransmitter usage. In our *in vitro* cultures, we found that the expression of glutamate decarboxylase *(GAD67)*, the enzyme that generates GABA from glutamate and glutaminase *(GLS)* that converts glutamine to glutamate, increase steadily from 14 DIV ( Figure 5 F, G). Finally, expression of the transporter vGLUT1 that mediates the uptake of glutamate from the synaptic cleft steadily increased from 21 DIV ( Figure 5 H).

We assessed the cellular composition of our *in vitro* differentiated cultures by quantifying the expression of markers for astrocytes (GFAP) and oligodendrocytes (OLIG2). The levels of GFAP transcripts were minimal prior to 21 DIV and then increased with the age of the culture ( Figure 5I) while that for OLIG2 was minimal until 45 DIV ( Figure 5J). GFAP staining was not detectable prior to 21 DIV, was minimal at 45 DIV and only increased from 60 DIV ( Figure 5K i, v, ix). OLIG2 protein expression was not detectable even at 60 DIV ( Figure 5K ii, vi, x). 

### Development of spontaneous neuronal activity *in vitro*

**Figure 6.**
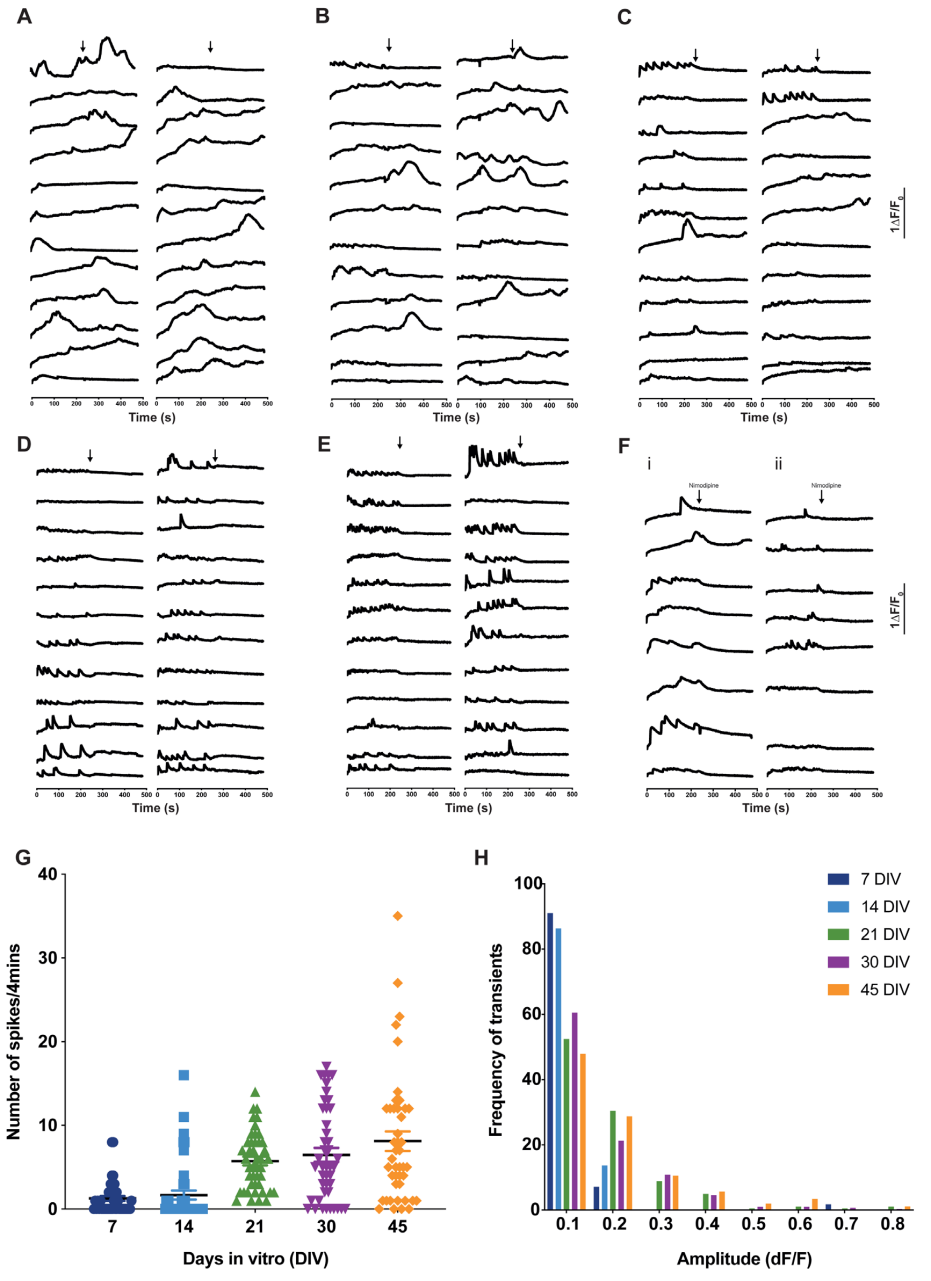
Development of Ca ^2+^ transients during *in-vitro* differentiation. ( **A**) to ( **E**) Calcium transients recorded at 7, 14, 21, 30 and 45 DIV are shown. Each panel shows [Ca ^2+^] _i_ traces from individual cells in the dish. Y-axis shows ΔF. X-axis is time in s. The baseline was recorded for 4 mins, followed by addition of 10μM tetrodotoxin (TTX) (as indicated by the arrows) to block Ca ^2+^ transients. ( **F**) Calcium transients recorded from 7 DIV (i) and 60 DIV (ii) cultures. Traces for individual cells are shown. An L-type VGCC blocker Nimodipine (10μM) was applied at the point indicated and completely abolished transients. ( **G**) The number of spikes/ 4 min was counted from individual neurons at each age and plotted. Symbols on the scatter-dot plot correspond to individual neurons. Mean± SEM for 30–40 neurons. ( **H**) Bar graph quantifying the amplitude of calcium transients that was analyzed and binned. X-axis shows the amplitude size of each bin. Y-axis shows the proportion of transients corresponding to that bin size at each age.

Spontaneous activity underpinned by changes in [Ca ^2+^] _i_ play a critical role in synaptic development, remodelling ^20^ and neurotransmitter choice ^21^ during neuronal development [reviewed in 22]. To measure spontaneous activity in our *in vitro* cultures, we measured fluctuations in [Ca ^2+^] _i_ *. In vitro* neuronal cultures at specified ages were loaded with fluorescent Ca ^2+^ indicators and examined for evidence of spontaneous elevations of [Ca ^2+^] _i_. Based on visual examination, two types of transients were noted: (i) those that were observed in neurites and (ii) those seen in the soma (Video 1, see extended data ^23^). In this study, we quantified [Ca ^2+^] _i_ transients that arose in the soma. We found that in 7 DIV neurons a minimal number of such transients were seen ( Figure 6A). The number of such transients progressively increased as a function of the age of the culture, i.e older cultures showed more transients/unit time ( Figure 6B-E, G). The resting membrane potentials (RMPs) of neurons during early stages of differentiation is reported to be depolarized and as neurons mature, their RMPs become hyperpolarized. This is validated by blocking the voltage-gated Na ^+^ channel using tetradotoxin (TTX). In our cultures, we observe that the activity displayed in the baseline measurement was abolished by addition of TTX starting 14 DIV onwards specifically in neurons that fired trains of spontaneous transients, indicating a mixed culture with some neurons maturing faster than rest. TTX did not completely abolish these transients in the early days of neuronal differentiation. In contrast, TTX abolished the calcium oscillations virtually in all 30 DIV and 45 DIV neurons. ( Figure 6A v 6D, E). A primary source of the spontaneous [Ca ^2+^] _i_ seen in our cultures is likely to be the activity of voltage activated Ca ^2+^ channels (VGCC). Consistent with this idea, we were able to abolish the [Ca ^2+^] _i_ transients by the application of the VGCC blocker, nimodipine ( Figure 6 F i, ii). Overall, we were able to maintain these cultures up to 120 DIV and observe [Ca ^2+^] _I_ transients at each age of assessment. However, in older cultures, cellular crowding occurs and it became progressively more difficult to unambiguously assign ROI to a single soma using epifluorescence microscopy. 

### Store operated calcium influx in developing neurons

**Figure 7.**
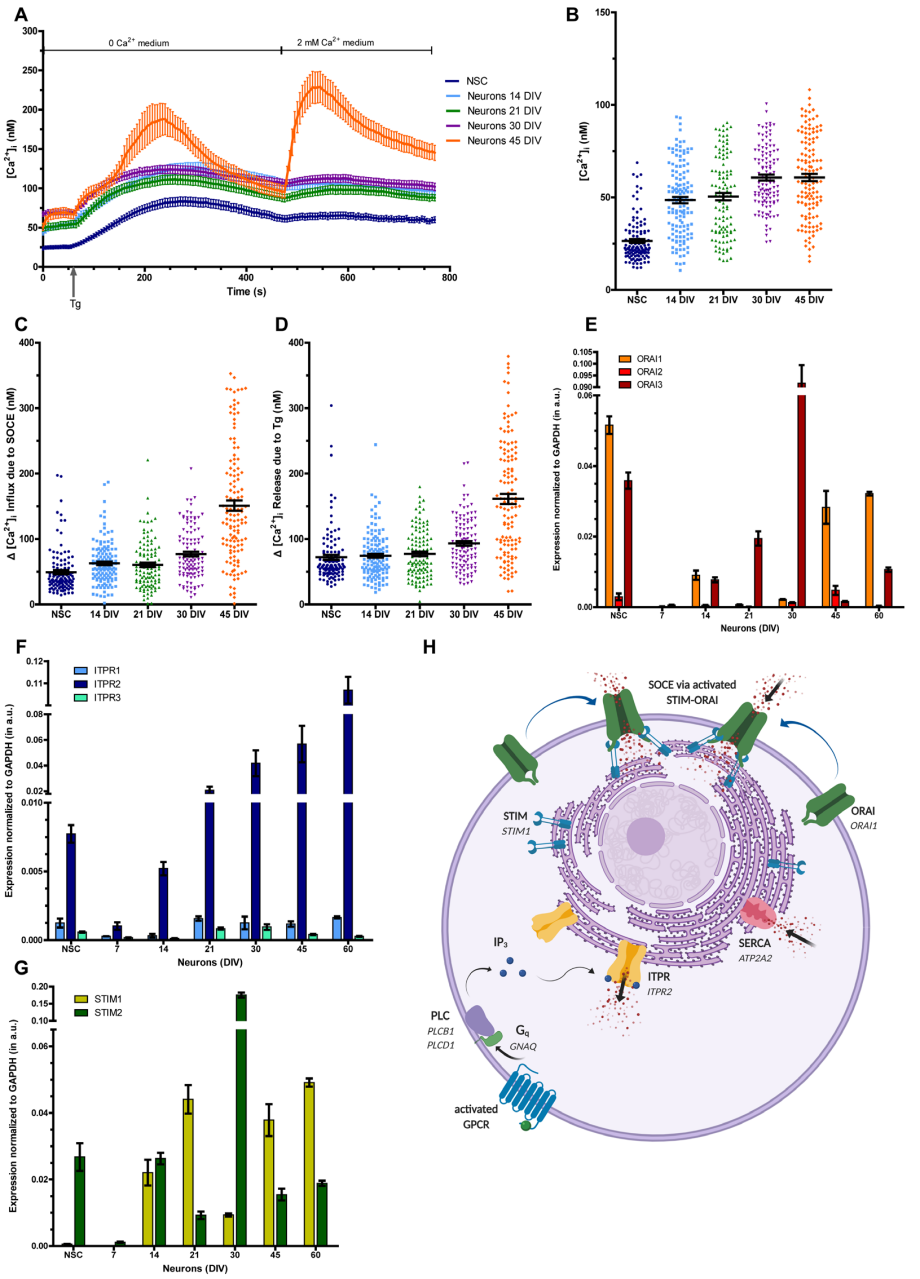
Characterization of store operated calcium entry (SOCE) in neural stem cells (NSCs) and *in vitro* differentiated cortical neurons ( **A**) [Ca ^2+^] _i_ levels measured using the ratiometric dye Fura-2 during Thapsigargin induced store release and subsequent SOCE in XCL1 NSCs and *in vitro* differentiated cortical neurons of specific ages. Each trace for a given age consists of the mean ± SEM for 25–30 cells. ( **B**) Scatter plot quantifying the resting cytosolic [Ca ^2+^] _i_; each point represents a single cell in the culture. X-axis shows age of neurons in culture (DIV). (C,D) Scatter plots quantifying [Ca ^2+^] _i_ rise above basal cytosolic [Ca ^2+^] _i_ for ( **C**) SOCE and ( **D**) Tg mediated store release respectively at various ages of *in vitro* culture. qPCR measurements to show the relative expression of various isoforms of ORAI ( **E**), STIM ( **G**) and the IP3 receptor (ITPR) ( **F**) at different stages of differentiation. ( **H**) Cartoon (created using Biorender) representing the components of SOCE that are expressed in differentiating NSC. Individual organelles and membranes are shown. The individual component of SOCE are shown in capital, bold. The genes predominantly encoding each component in these cells are shown in italics. (Abbreviations: Gq is G protein α-subunit, encoded by the gene GNAQ; PLC is Phospholipase C, the predominantly expressed isoforms are PLCβ _1_and PLCδ _1_, encoded respectively by PLCB1 and PLCD1; SERCA (Sarco/Endoplasmic Reticulum Ca ^2+^-ATPase) is encoded mainly by the gene ATP2A2.)

Many neuronal responses are modulated by the activation of cell surface receptors that couple to the G-protein subunit Gq, activating phospholipase C (PLC) and culminating in the influx of extracellular Ca ^2+^ through a process of SOCE. To test the presence of SOCE in our *in vitro* cultures, we performed experiments where we depleted internal Ca ^2+^ stores using the SERCA pump inhibitor thapsigargin and observed both intracellular release and the influx of Ca ^2+^ into the cytosol from the extracellular space. In our experiments we observed that the average resting [Ca ^2+^] _i_ was ca 26 nM; this rose to 48.5 nM in 14 DIV and was subsequently only slightly elevated thereafter up to 45 DIV ( Figure 7B). In the initial part of the experiment, we applied thapsigargin to cultures loaded with Ca ^2+^ indicator but bathed in zero extracellualr Ca ^2+^. The rise in fluorescence represents the release of Ca ^2+^ from intracellular stores and could be readily observed at all stages of differentiation ( Figure 7A). We quantified this release which approximates the size of the ER Ca ^2+ ^pool and noted that the smallest elevation of [Ca ^2+^] _i_ was seen in NSC and the largest in 45 DIV neurons ( Figure 7D). Finally we measured the peak of the [Ca ^2+^] _i_ following add back of extracellular Ca ^2+^ and found that while minimal SOCE was seen in NSC, significant influx was only seen in 45 DIV( Figure 7A, C). 

To understand the development of SOCE based Ca ^2+^ influx, we measured the expression of the core elements of the SOCE pathway ^24^. SOCE is mediated by the ORAI channel. Three genes encode this protein in the human genome. Of these *ORAI1* and *ORAI3* were expressed in NSC with minimal expression of *ORAI2*. During differentiation ORAI1 expression was enhanced at 45 DIV and while that of *ORAI3* at 30 DIV ( Figure 7E). The activation of ORAI is mediated by store depletion, triggered *in vivo* by inositol 1,4,5 trisphosphate (IP _3_) receptor mediated release of Ca ^2+^ from internal stores. This store depletion is sensed by the ER localized Ca ^2+^ sensor STIM which then leads to activation of ORAI. We measured the expression of IP _3_ receptor and STIM in our cultures. While *ITPR1* and *ITPR3* were expressed at negligible levels, *ITPR2* expression increased steadily during differentiation ( Figure 7F). Two genes encode for STIM in the human genome; of these *STIM1* which activates ORAI channels was expressed at very low levels in NSC and its expression increased steadily fom 14 DIV onwards ( Figure 7G). By contrast *STIM2* was expressed at low levels at all stages except at 30 DIV when its expression was substantially elevated ( Figure 7G). Interestingly, at exactly that stage the level of *STIM1* dropped significantly ( Figure 7G).

### Molecular basis of spontaneous Ca ^2+^ signals in developing neurons

A likely value of this experimental model is the ability to test for changes in Ca ^2+^ signalling in human patients with specific diseases of the brain using patient derived iPSC differentiated into neurons. In addition to the activity of VGCC, [Ca ^2+^] _i_ transients can also arise from the activity of N-methyl D-aspartate (NMDAR), Ca ^2+^ permeable AMPAR, nAChR and the shape, size and frequency of the transients can be modulated by expression of other components of the Ca ^2+^ signalling toolkit ^1^. Once changes in Ca ^2+^ signalling are detected in neurons differentiated from patient cells, a natural next step is to probe the molecular basis of such changes. Towards this end, it is necessary to have a description of the Ca ^2+^ signalling toolkit operating in neurons differentiated using this protocol so that observed changes may be mapped to specific molecular processes. To establish the likely molecular components that shape Ca ^2+^ transients in the developing *in vitro* neuronal cultures, we performed an RNA-seq analysis of transcripts from NSC, 45 DIV and 60 DIV neurons.

**Figure 8.**
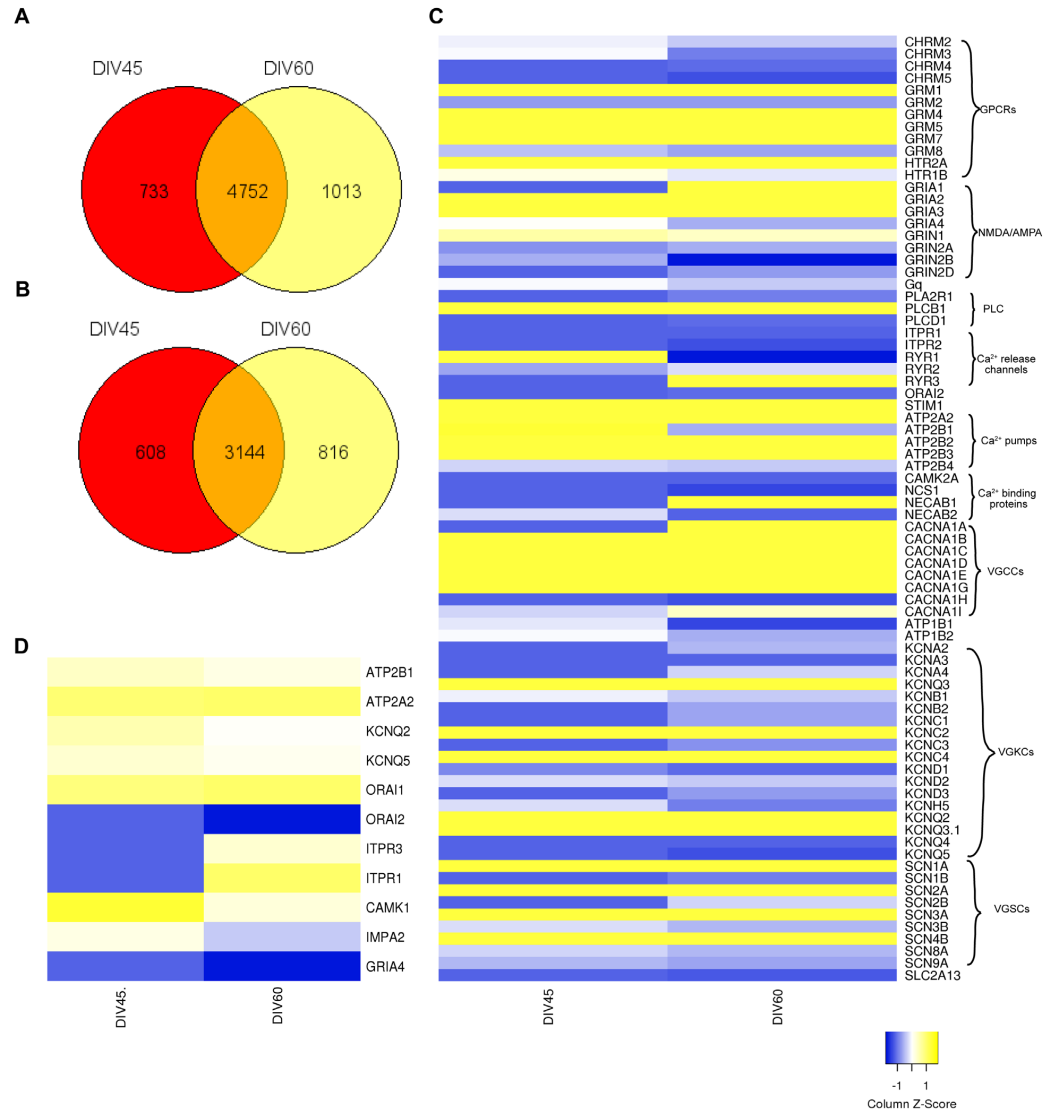
Heatmap to showing the calcium signalling transcriptome of neural stem cells (NSCs) and neurons at 45 days *in vitro* (DIV) and 60 DIV. ( **A**) Venn diagram to show the number of common upregulated genes between sample 45 DIV and 60 DIV: The total number of upregulated genes (log _2_ FC > +1.5) were counted in each sample and the number of common upregulated genes were calculated between 45 DIV and 60 DIV. These values have been plotted as Venn diagram. ( **B**) Venn diagram to show number of common downregulated genes between sample 45 DIV and 60 DIV: The total number of downregulated genes (log _2_ FC < -1.5) were counted in each sample and the number of common downregulated genes were calculated between 45 DIV45 and 60 DIV. These values have been plotted as Venn diagram. ( **C**) Upregulated genes: The heatmap represents the extent of upregulation of genes directly involved in calcium signalling in 45 DIV and 60 DIV samples compared to NSCs. The values have been scaled by column Z-score and scale ranges from -1 to +1 (blue to yellow). The gene names as from HUGO nomenclature are represented on Y-axis. The heatmap was made using heatmappeR. The genes have been grouped into the following categories: GPCRs (G-Protein Coupled Receptors), AMPA/NMDA receptors, PLC (Phospholipases), Ca ^2+^ release channels, Ca ^2+^ pumps, Ca ^2+^ binding proteins, VGCCs (Voltage-Gated Calcium Channels), VGKCs (Voltage-Gated Potassium Channels) and VGSCs (Voltage-Gated Sodium Channels). ( **D**) Downregulated genes: The heatmap represents the extent of downregulation of genes involved in calcium signalling in 45 DIV and 60 DIV samples compared to NSCs. The values have been scaled by column Z-score and scale ranges from -1 to +1 (blue to yellow). The gene names as from ensemble nomenclature are represented on Y-axis. The heatmap was made using heatmappeR.

We found ca. 6000 upregulated and ca. 3700 downregulated transcripts in 45 DIV (See underlying data) and 60 DIV neurons (See underlying data) compared to NSC. The vast majority of these altered transcripts were common to both 45 and 60 DIV neurons ( Figure 8A, B) although a few were specifically changed in either 45 DIV or 60 DIV only. We curated a list of genes encoding molecules likely to directly regulate Ca ^2+^ signalling. These are indicated in Figure 8C (upregulated genes) and Figure 8D (downregulated genes). We found that of the many possible genes in the human genome that could encode a specific component, transcripts for only a subset were expressed in 45 DIV or 60 DIV neurons; for example, of the 10 genes that encode different subunits of VGCC in the human genome, only six genes were expressed in differentiated neurons at 45 DIV and 60 DIV. Two of them were expressed only in 60 DIV and one of them was highly upregulated in 60 DIV as compared to 45 DIV ( Figure 8C). Thus, analysis of VGCC activity and its role in mediating any Ca ^2+^ signalling phenotype can now be focused to these genes, for example by RNAi mediated depletion or CRISPR/Cas9 gene editing.

## Discussion

Brain structure and function can be investigated at multiple scales of analysis including behavioural output, structural and functional imaging (e.g MRI) and clinical electrophysiology and these have been extensively used in the analysis of brain disease biology. However, a long-standing challenge in studying human brain disease is the ability to study mechanisms at the level of cellular and molecular processes in living human brain cells that typically require neural cells in a dish. The advent of modern stem cell technology that allows the generation of iPSC from the somatic cells of humans and their subsequent differentiation into neural cell types *in vitro* offers a solution to this problem ^8^. The human brain consists of multiple cell types including neurons, glial cells and vasculature. In additions, for a single cell type such as neurons, each region of the brain contains neurons of distinct molecular identities and physiological properties. To faciliate this, we have developed a protocol that allows human cortical neurons to be differentiated starting with NSC, a stem cell population observed during brain development ^25^. Our protocol described here allows developing neurons to be maintained in culture for almost 120 DIV. This corresponds to 15—16 post-conception weeks of the human brain development ^26^. These cultures can be studied using live imaging methods to observe and quantify cell biological and physiological processes upto this age *in vitro* and thus allow the reconstruction of physiological development of these neurons.This strategy will allow the comparison of neuronal development between NSC derived from normal human subjects as well as those derived from patients with specific brain disorders. Given the proposed developmental origins of many human brain diseases ^27^, our approach will allow the analysis of defects in cell physiology in developing neurons.

The human cerebral cortex is a complex cellular structure composed of six layers each of which contains specilized neuronal types with distinct functional properties and projections. In using *in vitro* cultures to study the properties of human brain cells differentiated from NSCs, it is necessary to establish the extent to which these cultures differentiate to form the cell types typical of the human cerebral cortex. The cell types of the cerebral cortex are specified by trancription factors that are typical for particular cell types or layers. Using qRT-PCR and immunocytochemistry, we found that our cultures show progressively increasing expression of Cut like homeobox 1 (CUX) [required for dendritic morphogenesis in layer II-III cortical neurons], CTIP2 a transcription factor typical of layers V-VI and *SATB2* of cortical layer II-IV. These findings suggest that our protocol generates *in vitro* cultures with cell types representative of several key layers of the human cerebral cortex. Thus, the physiological properties of the neurons generated by this method are likely to reflect at least some of the properties of neurons in the human cerebral cortex. In this respect, these culture will likely be useful for the analysis of physiological changes in human brain disorders where the cerebral cortex is primarily affected. These include several key examples of severe mental illness including schizophrenia, bipolar disorder, neurodevelopmental disorders that affect cortical development and function as well as neurological disorders such as epilepsy.

In this study, we have imaged and quantified the frequency and amplitude of [Ca ^2+^] _i_ transient development. We found that in our culture conditions, the frequency of [Ca ^2+^] _i_ transients increased as a function of the age of the culture (DIV) ( Figure 6 G,H). The [Ca ^2+^] _i_ transients were blocked by the addition of nimodipine, a blocker of VGCC suggesting that the activity of VGCC underlies their generation. In addition, the [Ca ^2+^] _i_ were also abolished by the application of TTX, a blocker of Na ^+^ channels suggesting that voltage changes presumably through the depolarization of neurons likely activates VGCC resulting in the [Ca ^2+^] _i_ observed by imaging. Overall these findings imply the progressive development of neuronal activity in the *in vitro* cultures as a function of age that can be monitored using Ca ^2+^ imaging. Thus our culture system and the use of [Ca ^2+^] _i_ transient imaging is likely to be of value in observing altered development of neuronal activity in cell lines derived from patients with specific brain disorders. In addition, through DNA sequencing, a number of mutations have been described in genes encoding components of the Ca ^2+^ signalling machinery in human patients. For example, numerous mutations have been described in CACNA1B (calcium channel, voltage-dependent, N-type, alpha 1B subunit) among patients with bipolar disorder, autism spectrum and Timothy syndrome ^7^. Interestingly, a recent study has described a role for VGCC in the development of mouse cortical neurons through the control of cellular processes such as neurite outgrowth and radial migration ^28^. The methods described in this paper will allow the impact of human mutations in calcium signalling genes (such as CACNA1B) to be studied in the context of neuronal development and physiology.

In addition to VGCC, many other mechanisms mediate changes in [Ca ^2+^] _i_. SOCE is one of these processes and is triggered by G-protein coupled PLC activation, a signalling mechanism used by several cell surface receptors that are expressed in the human cerebral cortex and reported to be important for brain function [e.g metabotropic glutamate receptor] and PLC signalling has also been linked to several brain disorders ^29^. Studies in murine neural precursor cells (NPCs) have shown robust SOCE and depletion of STIM1 or ORAI1, key elements of SOCE results in defective NPC proliferation ^30^; a recent study has also suggested a conserved role for SOCE in human NPC proliferation ^31^. In this study we developed protocols to monitor SOCE during *in vitro* differentiation and found a dynamic pattern of SOCE activity. Thus, our culture system and imaging protocols can be used to study the role of SOCE in cells derived from patients with specific human brain diseases. In addition, PLC signalling also triggers phosphoinositide turnover and the mechanism of action of lithium, used in the management of bipolar disorder, is thought to occur via the modulation of inositol recycling in this setting [reviewed in 32]. This question can therefore be analysed using the protocols described here coupled with the analysis of phosphoinositide turnover using biochemical approaches.

Human brain diseases may arise from multiple mechanisms including both cell-autonomous processes in neurons and also as an outcome of neuronal dysfunction arising through cell-cell interaction mechanisms between neurons and other cell types. Regardless of these different mechanisms, studying any given cell-biological process in human neurons requires the generation of *in vitro* neuronal live cultures in which physiological processes can be studied in the context of neuronal development. In our protocol, we found that there was minimal presence of non-neuronal cells until ca. 21 DIV but from 30 DIV transcripts for GFAP increased and GFAP immunoreactivity was seen from 45 DIV onwards. This time course of gliogenesis observed in our culture system is consistent with previous studies that have reported a substantial increase in GFAP+ cells over time following the initial appearance of neuronal cells during brain development both *in vivo* ^33^ and *in vitro* ^34^. Thus upto ca. 30 DIV our cultures will be suitable for the analysis of cell-autonomous phenotypes arising from neurons but thereafter, phenotypes observed may reflect contributions from both neurons and glial cells present in the cultures. Interestingly, an increasing number of brain diseases are linked to neuron-glia interactions ^35, 36^ and could be studied in older cultures of the type we observed post 30 DIV.

In summary, we have developed a protocol that allows the quantitative analysis of Ca ^2+^ signalling defects in neural cells in the context of human diseases that affect the brain. The use of this approach should provide mechanistic insights into the cell biology and physiology of human neurodevelopmental disorders and may further lead to the development of therapeutic approaches for these conditions.

## Data availability

### Underlying data

Open Science Framework: Calcium imaging data_Sharma et.al.2019_NCBS TIFR. https://doi.org/10.17605/OSF.IO/V7XE6 ^23^

This project contains the following underlying data:

○ Figure panel 1
  ○ Cell proliferation data.xlsx (growth curve of XCL1 NSCs)
  ○ Figure 1C (Folder containing Immunofluorescence confocal images for characterization of neural stem cell markers)
  ○ Karyotype report.pdf (cytogenetic characterization of XCL1 NSCs)
○ Figure panel 3 (Folder containing immunofluorescence confocal images of neuronal markers)
○ Figure panel 4 (Folder containing Immunofluorescence confocal images of cortical layer marker)
○ Figure panel 5 (Folder containing the confocal images listed below)
  ○ Figure 5D,E (Immunofluorescence confocal images of synaptic markers)
  ○ Figure 5K (Immunofluorescence confocal images of glial markers)
  ○ Figure panel 6 (Folder containing Excel files for Ca ^2+^ imaging for Ca ^2+^ transient activity)
○ Figure panel 7 (Folder containing Excel files for SOCE characterization)
○ qRT-PCR data
  ○ Ct values for qRT-PCR experiments.xlsx (Excel file containing raw Ct values for qRT-PCR)
  ○ ITPR neurons expression qpcr raw ct values.xlsx (Excel file containing raw Ct values for qRT-PCR measuring ITPR expression)
  ○ STIM and ORAI expression qpcr raw ct values.xlsx (Excel file containing raw Ct values for qRT-PCR measuring STIM and ORAI expression)

### Extended data

Open Science Framework: Calcium imaging data_Sharma *et al.* 2019_NCBS TIFR. https://doi.org/10.17605/OSF.IO/V7XE6 ^23^

This project contains the following extended data:

- Sharma_Video1.avi (Calcium imaging recordings of 45 DIV cortical neurons showing spontaneous activities in the soma as well as the neurites. Cells were loaded with Fluo-4/AM and imaged at 1 fps for 480 s)

Data are available under the terms of the Creative Commons Zero "No rights reserved" data waiver (CC0 1.0 Public domain dedication).

RNA sequencing data has been submitted to the Sequence Read Archive database on NCBI under project ID PRJNA600215.

RNA-seq of neurons, Accession numner SRX7527467: https://identifiers.org/insdc.sra:SRX7527467

RNA-seq of XCL1, Accession number SRX7527466: https://identifiers.org/insdc.sra:SRX7527466
