# Supplementary material for: In vitro human stem cell derived cultures to monitor calcium signaling in neuronal development and function
Source: Wellcome Open Res. 2020 Feb 3;5:16. [Version 1] doi: 10.12688/wellcomeopenres.15626.1 (PMC7076282; doi:10.12688/wellcomeopenres.15626.1)
Supplement: Peer review history

# Peer review: Reviewer response for version 1

**DOI:** 10.21956/wellcomeopenres.17119.r37771

**Published:** 2020-03-23

**Authors:** Murali Prakriya

**Affiliations:** 1 Department of Pharmacology, Feinberg School of Medicine, Northwestern University, Chicago, IL, USA

This is a nice review of procedures for differentiating neural progenitor lines into neurons and a careful description of the molecular and physiological properties of the differentiated neurons as they go through the differentiation process. It is well-written, very detailed, and will be useful for several investigators interested in the field as well as also providing a general list of tools, reagents, and procedures that could be applied more generally to workers in other related areas. I only have a few minor comments and suggestions.

  

1. Page 9. In the Grynkiewicz equation, the term ca2+, the “C” should be capitalized. 
2. Related, it is curious that the authors chose to use a Kd of 225 nM. Many other labs use an in vitro value of 135 nM. Please provide a citation for the use of 225 nM. 
3. In Figure 6A-F, it is relatively hard to see how the arrow (indicating time of application) of TTX corresponds to the traces at the bottom of each panel. A dotted line going through all the traces in each panel would provide better clarity. 
4. Likewise, in Figure 7A, please show the application of thapsigargin at the top, along the bars showing 0 mM Ca/2 mM Ca etc. It is hard to see the arrow at the bottom axis. 
5. Page 19, second paragraph on the left, 2 ^nd^ line. specilizedà spealized. Typo. 

I confirm that I have read this submission and believe that I have an appropriate level of expertise to confirm that it is of an acceptable scientific standard.

# Peer review: Reviewer response for version 1

**DOI:** 10.21956/wellcomeopenres.17119.r38053

**Published:** 2020-03-13

**Authors:** Laura N. Borodinsky

**Affiliations:** 1 Department of Physiology and Membrane Biology, Institute for Pediatric Regenerative Medicine, Shriners Hospital for Children, School of Medicine, University of California, Davis, Sacramento, CA, USA

The manuscript by Sharma *et al.* “ **In vitro human stem cell derived cultures to monitor calcium**

**signaling in neuronal development and function**” provides a methodological resource to assess calcium dynamics in human iPSC-derived cultured neurons. This approach might enable the modelling of human neurological diseases, specifically neurodevelopmental disorders, given that these cultures can be maintain for up to 120 days, which corresponds to 15-16 weeks of gestation.   

 This is a useful resource that may be of interest to diverse fields including, developmental biology and neuroscience, stem cell research and neurophysiology.

 There are some specific issues that should be addressed:

  

1. In Methods, section “ *E) Calculation of cell proliferation rate*”, “the authors proposed to count cells to determine proliferation rate. This is inadequate, number of cells can change by differences in proliferation rate and differences in cell survival. The authors will not be able to distinguish between these two cellular events from counting cells. More specialized approaches are required to sort these two cellular processes out.
2. GFAP and OLIG2 are not exclusive markers for astrocytes and oligodendrocytes, respectively. The authors should acknowledge the limitations of using these markers for identifying these cell types. 
3. Page 13, 1 ^st^ column, last sentence should be revised: “ *Finally, expression of the transporter vGLUT1 that mediates the uptake of glutamate from the synaptic cleft steadily increased from 21 DIV (Figure 5 H)*”. This is wrong, VGLUT1 uptakes glutamate into vesicles inside the presynaptic glutamatergic neuron to enable release upon stimulation.
4. Figure 6H should be removed. A non-ratiometric dye like Fluo4-AM does not really allow for analysis of calcium transient amplitudes in different samples/culture dishes because of the variability in the loading of the dye in different cultures. The differences observed might not reflect the actual magnitude of changes in [Ca ^2+^] _I_, but instead represent a technical difference in the loading of the dye in different cultures of different age, etc.
5. Figure 8C,D. Why are there some genes (ORAI2, ITRP1) in both C and D if one shows the upregulated (C) and the other the downregulated genes (D)?
6. “ *Thus upto ca. 30 DIV our cultures will be suitable for the analysis of cell-autonomous phenotypes arising from neurons but thereafter, phenotypes observed may reflect contributions from both neurons and glial cells present in the cultures*”. The authors should revise the “cell-autonomous” term by replacing it with neuronal-specific or cell-type specific. It could be non-cell autonomous even if glia cells are not participating, if, for instance is through neuronal connections or neuronal release of some factors that affect phenotypes in a non-cell-autonomous manner.

I confirm that I have read this submission and believe that I have an appropriate level of expertise to confirm that it is of an acceptable scientific standard.

# Peer review: Reviewer response for version 1

**DOI:** 10.21956/wellcomeopenres.17119.r37774

**Published:** 2020-03-11

**Authors:** Mohan C. Vemuri

**Affiliations:** 1 Thermo Fisher Scientific, Frederick, MD, USA

The paper describes an in vitro human neuronal culture system for the study of calcium signaling in normal and patients with any brain disorders. These cultures can be extended up to 120 days in a culture dish - which is a huge advantage to researchers.

 The description of the method for others to follow and repeat is well laid.

 Methodology of neural differentiation, characterization with specific NSC and NPC and non-neural cell markers and transcriptome analysis to pin point the cluster of genes that are likely impacted is done in an exceptionally nice and convincing way.

 Few questions:

1. Should Sox1 staining not be generally nuclear? I see it looks more cytoplasmic.
2. This study is conducted with only XCL1 neural stem cells from commercial sources. Although its nice and good, it would have been better to include a disease based NSC (commercial or developed institutionally) and validate these methods for disease paradigms.

Authors - please do not consider this comment of mine as negative.

 I really commend the authors and investigators to develop this robust method that will be useful for all global researchers.

I confirm that I have read this submission and believe that I have an appropriate level of expertise to confirm that it is of an acceptable scientific standard.
